# Progression of patellofemoral joint cartilage degeneration within 1 year after medial meniscus posterior root repair: A retrospective study

**DOI:** 10.1002/jeo2.70139

**Published:** 2025-04-01

**Authors:** Masanori Tamura, Takayuki Furumatsu, Yusuke Yokoyama, Yuki Okazaki, Koki Kawada, Tsubasa Hasegawa, Toshifumi Ozaki

**Affiliations:** ^1^ Department of Orthopaedic Surgery Okayama University Graduate School of Medicine, Dentistry, and Pharmaceutical Sciences Okayama Japan; ^2^ Department of Orthopaedic Surgery Japanese Red Cross Okayama Hospital Okayama Japan

**Keywords:** medial meniscus, posterior root tear, pullout repair, rehabilitation, second‐look arthroscopy

## Abstract

**Purpose:**

To assess postoperative progression of patellofemoral (PF) cartilage degeneration after medial meniscus posterior root (MMPR) repair and identify potential risk factors.

**Methods:**

Data from patients who underwent transtibial pullout repair for complete radial MMPR tears between April 2018 and October 2021 were retrospectively investigated. Patients with severe chondral lesions of the PF joint at primary surgery were excluded. All patients underwent second‐look arthroscopy at 12 months postoperatively. Postoperative changes using the International Cartilage Repair Society (ICRS) grade were evaluated. Associated open magnetic resonance imaging (MRI) findings were assessed.

**Results:**

In total, 40 patients (30 women, 10 men; mean age: 64.0 years) were evaluated. PF joint cartilage degeneration progressed significantly postoperatively. Abnormal signal intensity (ASI) of the infrapatellar fat pad (IPFP) was observed in 15 (37.5%) patients. Arthroscopic findings in groups between IPFP with and without ASI were compared. The incidence of postoperative ICRS grade worsening (≥2 grades) on the patella or trochlea was significantly higher among patients with ASI (53%) than among those without (20%, *p* = 0.04). ICRS grade worsening in the medial femorotibial compartment and meniscus‐healing status were comparable between the groups. Patients with ASI of the IPFP showed greater decrease in the distance between the patellar and anterior cruciate ligament insertions on knee flexion MRI (−1.5 ± 0.7 mm) than that in those without (−0.2 ± 0.3 mm, *p* < 0.01). A delayed rehabilitation protocol was a risk factor according to the logistic regression analysis (*p* = 0.01).

**Conclusions:**

Progressive PF cartilage degeneration occurred following MMPR repair, highlighting the need for diligent postoperative PF joint management.

**Level of Evidence:**

Level IV case series.

**Abbreviations**
ACL: anterior cruciate ligament
ASI: abnormal signal intensity
BMI: body mass index
ICRS: International Cartilage Repair Society
IPFP: infrapatellar fat pad
KOOS: Knee Injury and Osteoarthritis Outcome Score
MMPR: medial meniscus posterior root
MMPRT: medial meniscus posterior root tear
MRI: magnetic resonance imaging
OA: osteoarthritis
PAL: patellar‐anterior cruciate ligament insertion length
PF: patellofemoral
PL: patellar length
PTL: patellar tendon length
ROM: range of motion
VAS: visual analogue scale

## INTRODUCTION

Medial meniscus posterior root (MMPR) tears (MMPRTs) occur as degenerative tears in middle‐aged or older patients, accounting for approximately 20% of all meniscal tears. These tears can induce the rapid progression of knee osteoarthritis (OA) of the medial femorotibial compartment with meniscal extrusion [11, 23, 26]. There has been a paradigm shift in the surgical treatment of MMPRTs. Partial meniscectomy used to be a common surgical procedure; however, recently, MMPR repairs, including transtibial pullout repair, have been increasingly performed. Compared with partial meniscectomy or conservative treatment, MMPR repairs aim to restore meniscal hoop tension, slow the progression of medial femorotibial OA and prevent arthroplasty conversion [3, 6, 11, 16, 17, 28, 32].

Recognizing complications is important for evaluating and enhancing treatment effectiveness. A recent systematic review of MMPR repair complications revealed the following common complications: progression of degenerative changes within the medial compartment (10.4%), conversion to total knee arthroscopy (1.3%), repair failure (3.1%) and persistent postoperative knee pain (3.2%) [12]. While previous studies have focused on meniscal healing status and the chondral lesions in the medial femorotibial joint, there is a lack of research on other abnormalities in the knee joint, including fibrosis of the infrapatellar fat pad (IPFP). Fibrosis of IPFP can cause knee stiffness, anterior knee pain and progression of patellofemoral (PF) degeneration after knee surgery [25, 29]. Recent reports have indicated that patients with degenerative MMPRT frequently have some cartilage lesions in the PF joint [19, 24]. However, the progression of PF joint degeneration after MMPR repair in patients without severe PFOA has seldom been reported. Postoperative PF chondral damage can be a hidden complication after MMPR repair, and care of the PF joint is important after the procedure.

This study aimed to determine whether PF cartilage degeneration progresses after MMPR repair in middle‐aged and older patients. We investigated imaging findings suggestive of PF cartilage degeneration progression and examined risk factors for abnormal signal intensity (ASI) in the IPFP in patients with MMPR repairs.

## METHODS

### Patients

The Institutional Review Board of Okayama University Hospital approved this retrospective study (approval number: N1857). The study included all patients who underwent transtibial pullout repair for complete radial MMPRTs (LaPrade type 2 tear) between April 2018 and October 2021, pre‐ and postoperative open magnetic resonance imaging (MRI) evaluation, and arthroscopic second‐look evaluation at 1 year postoperatively. Transtibial pullout repair was indicated in patients with the following criteria: continuous knee pain, femorotibial angle ≤180°, radiographic Kellgren–Lawrence grade 0–2 without subchondral insufficiency fractures, mild cartilage lesions (International Cartilage Repair Society [ICRS] grade ≤ 2) in the medial femorotibial compartment, and body mass index (BMI) < 35 kg/m^2^. The study exclusion criteria were the presence of a severe chondral lesion (ICRS grade ≥3) of the PF joint at the time of primary surgery and arthroscopic treatment for postoperative arthrofibrosis or range of motion (ROM) restriction.

### Surgical techniques

All surgical procedures were performed by the same orthopaedic surgeon (T. F.). The following four different suture configurations were used: two‐simple‐stitch using No. 2 polyethylene sutures, two‐simple‐stitch with an additional posteromedial pullout technique, two‐cinch stitch using No. 2 polyethylene sutures, and two‐cinch stitch with an additional posterior anchoring technique. A tibial tunnel was created using dedicated aiming devices. The pullout sutures were fixed on the tibia using a bioabsorbable interference screw and tied under an anchor screw at a knee flexion angle of 30° with an initial tension of 10–30 N.

### Postoperative rehabilitation protocols

Two different postoperative rehabilitation programmes were performed according to the date of surgery (Supporting Information S1: Table 1). Before May 2019, patients were nonweight‐bearing and required to wear a knee immobilizer for 2 weeks postoperatively. ROM exercises were initiated starting at 30° of knee flexion and gradually increased ( +30°/week) to 120°. Full ROM was allowed at 3 months postoperatively. Partial weight‐bearing of <20 kg was initiated at 2 weeks postoperatively, and weight‐bearing was increased by 20 kg weekly to full weight‐bearing according to the patient's weight. After May 2019, patients utilized a knee immobilizer for 1 week, and ROM exercises and partial weight‐bearing were initiated 1 week postoperatively, aiming for early recovery after MMPR repair. ROM exercises were initiated starting at 30° of knee flexion and gradually increased (+30°/week) to 120°. Full ROM was allowed at 2 months postoperatively. Partial weight‐bearing of <20 kg was permitted at 1 week postoperatively, and weight‐bearing was increased by 20 kg weekly to enable full weight‐bearing according to the patient's weight. Under both rehabilitation protocols, partial weight loading was controlled using a scale, and patients were advised to avoid knee hyperflexion in weight‐bearing situations, such as squatting, even after meniscal healing. Most patients remained in hospital until they could walk freely without external aids. After returning home, supervised rehabilitation was recommended two times a week for 2–3 months postoperatively.

### Methods of assessment

**Figure 1:**
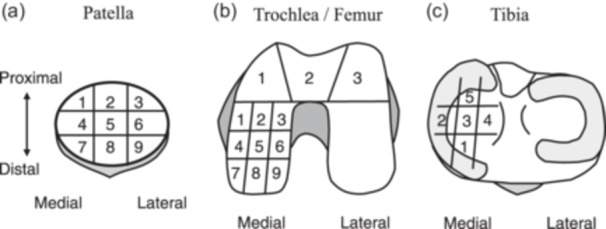
Schematic illustrations of the segmentation of the knee cartilage in arthroscopic assessment. (a) The patella was divided into nine segments. (b) The trochlea was divided into three segments, and the medial femoral condyles were divided into nine segments. (c) The medial tibial plateau was divided into five segments.

The degree of cartilage damage in the patella, trochlea, medial femoral condyle (MFC) and medial tibial plateau (MTP) was assessed using the ICRS grade classification [5] during primary surgery and second‐look arthroscopy. The patella and MFC were divided into nine zones, the MTP into five zones, and the trochlea into three zones (Figure 1). The ICRS grade changes in each area were compared between primary surgery and second‐look arthroscopy.

During second‐look arthroscopy, the meniscal healing score, comprising three subscales (anteroposterior width of bridging tissues, stability and synovial coverage [9]), was evaluated.

**Figure 2:**
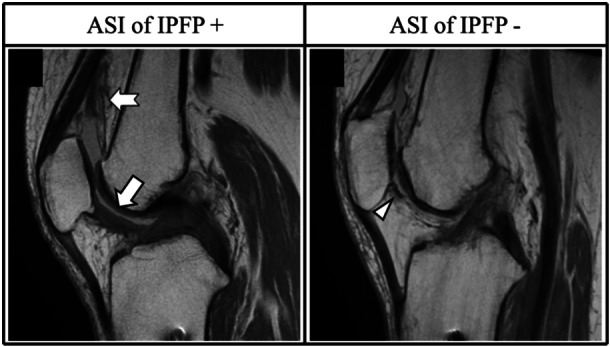
Magnetic resonance imaging findings of abnormal signal intensity (ASI) of the infrapatellar fat pad. (a, b) Sagittal images of the knee flexed at 10° at 1 year postoperatively. (a) Continuous increased low signal intensity between the patella and anterior cruciate ligament insertion (arrow). Mostly concomitant ASI of the suprapatellar pouch was found (swallow‐tail arrow). (b) Focal area of low signal intensity (arrowhead).

**Figure 3:**
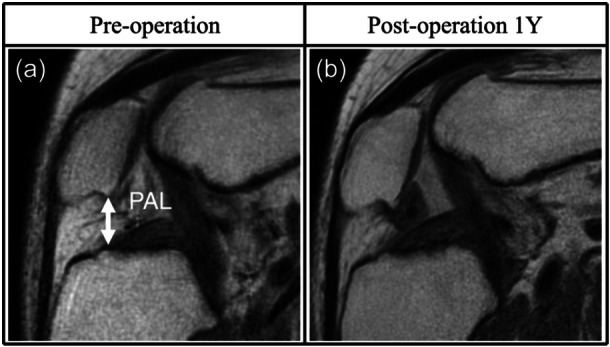
Magnetic resonance imaging findings of the postoperative reduction in patellar‐anterior cruciate ligament insertion length (PAL). (a, b) Sagittal images of the knee flexed at 90°. (a) PAL was defined as the length between the inferior edge of the patellar subchondral bone and the anterior cruciate ligament insertion, excluding osteophytes (length of the double‐headed arrow). (b) The PAL shortened postoperatively compared with that preoperatively.

Open MRI scanning was conducted preoperatively and 1 year postoperatively using an Oasis 1.2 T device (Hitachi Medical) with a coil in the 10° and 90° knee‐flexed positions under nonweight‐bearing conditions. Standard MRI sequences were obtained using a three‐dimensional sagittal proton density‐weighted sequence with a driven equilibrium pulse and a 90° flip angle. The repetition time/echo time was 500/120 and 600/96 for the 10° and 90° knee‐flexed positions, respectively. The slice thickness was 1 mm with no gap. The field of view was 18 cm, and the acquisition matrix size was 224 (phase) × 224 (frequency). If a continuous low signal intensity bridged the patella and the anterior cruciate ligament (ACL) insertion, the ASI of the IPFP was defined as positive. Conversely, if the low signal intensity in the IPFP was focal, the ASI was considered negative (Figure 2). The ASI of the suprapatellar pouch was also assessed, and it was considered positive if diffused in the superior half of the suprapatellar pouch. The Insall‐Salvati score was evaluated according to a previous report [27]. None of the patients presented with patella alta (Insall‐Salvati > 1.2) or patella baja (Insall‐Salvati < 0.8). The patellar‐ACL insertion length (PAL) was assessed at 90° of knee flexion (Figure 3) and the postoperative change (ΔPAL) was evaluated. Patellar tilt was assessed at 10° of knee flexion, as previously reported, and the postoperative chance (Δ patellar tilt) was evaluated [10].

Clinical scores were evaluated during primary surgery and second‐look arthroscopy using the Knee Injury and Osteoarthritis Outcome Score (KOOS) and a visual analogue scale (VAS) with pain scores ranging from 0 (no pain) to 100 mm (worst pain).

### Statistical analysis

Statistical analyses were performed using EZR software (Saitama Medical Centre, Jichi Medical University). Statistical significance was set at *p* < 0.05. The Kolmogorov–Smirnov test categorized each parameter as a parametric distribution for patient characteristics and MRI findings such as patellar tendon length, Insall‐Salvati and PAL and as a nonparametric distribution for cartilage status (ICRS grade) in arthroscopy and clinical scores.

Differences in cartilage damage between primary surgery and second‐look arthroscopy were assessed using Wilcoxon's signed‐rank test. The Mann–Whitney U test was used to compare the averages of continuous variables (such as age) and Fisher's exact test was used to evaluate the proportions of categorical variables (such as sex) between patients with and without ASI of the IPFP. Binomial logistic regression analysis was performed to analyze risk factors related to ASI in the IPFP. The independent variables of age, sex, BMI, rehabilitation protocol (initial protocol before May 2019 [the 2‐week protocol] or later protocol after May 2019 [the 1‐week protocol]), and preoperative Insall‐Salvati score were assessed, as reported previously [21, 29]. Intraobserver and interobserver correlations were assessed using intraclass correlation coefficients (ICC). A post hoc analysis using G*power 3.1 (Heinrich‐Heine‐Universität) showed that the sample size of 35 in this study was sufficient to achieve a statistical power of 80% (α = 0.05).

## RESULTS

Of the 45 patients who underwent surgery, five were excluded (four patients with severe chondral lesions at the PF joint at the time of primary surgery and one with additional arthroscopic treatment for arthrofibrosis 3 months after primary surgery). Ultimately, 40 patients were enroled in this study. Patient clinical characteristics are summarized in Table 1.

**Table 1:** Patient demographics and clinical characteristics.

Number of patients	40
Sex, male/female	10/30
Age, y (range)	64.0 ± 8.7 (57–71)
Height, m (range)	1.57 ± 0.1 (1.51–1.63)
Weight, kg (range)	62.1 ± 9.9 (54.0–69.8)
Body mass index, kg/m^2^ (range)	25.2 ± 2.5 (23.4–26.5)
Femorotibial angle, deg (range)	177.5 ± 2.3 (176–179)
Preoperative Kellgren–Lawrence grade, 0:1:2	0:21:19
Duration from injury to operation, day (range)	77.0 ± 59.6 (36–117)
Surgical technique (TSS/TSS + PM/TCS/TCS + PA)	10/9/9/12

*Note*: Values are presented as mean ± standard deviation or number. Range data are presented as first‐third quartiles.

Abbreviations: PA, posterior anchoring; PM, posteromedial pullout; TCS, two‐cinch stitches; TSS, two simple stitches.

Significant cartilage worsening between primary surgery and second‐look arthroscopy was observed in the patella (areas 1, 2, 4, 5 and 7), trochlea (area 2) and MFC (area 7), although no significant difference was observed in the MTP (Tables 2 and 3).

**Table 2:** Differences in the cartilage status (ICRS grade) between primary and second‐look arthroscopy.

	Patella		
Area	Primary	Second look	*p* Value
**1**	**1.5** ± **0.6**	**1.8** ± **0.4**	**<0.01** *
**2**	**1.5** ± **0.7**	**1.9** ± **0.4**	**<0.01** *
3	1.1 ± 0.6	1.2 ± 0.5	n.s.
**4**	**1.4** ± **0.6**	**1.9** ± **0.3**	**<0.01** *
**5**	**1.7** ± **0.6**	**2.0** ± **0.1**	**<0.01** *
6	1.0 ± 0.6	1.0 ± 0.5	n.s.
**7**	**1.2** ± **0.7**	**1.6** ± **0.6**	**<0.01** *
8	0.9 ± 0.8	1.1 ± 0.7	n.s.
9	0.5 ± 0.8	0.7 ± 0.7	n.s.

	Trochlea		
Area	Primary	Second look	*p* Value
1	1.7 ± 0.7	1.9 ± 0.4	n.s.
**2**	**1.7** ± **0.6**	**2.0** ± **0.3**	**<0.05** *
3	0.3 ± 0.6	0.2 ± 0.6	n.s.

*Note*: Data are displayed as a mean ± standard deviation. The significance was determined with the use of the Wilcoxon signed‐rank test.

Abbreviations: ICRS, International Cartilage Research Society; n.s., not significant.

* Values in bold indicate statistical significance (*p* < 0.05).

**Table 3:** Differences in the cartilage status (ICRS grade) between primary and second‐look arthroscopy.

	Medial femoral condyle		
Area	Primary	Second look	*p* Value
1	1.7 ± 0.5	1.8 ± 0.4	n.s.
2	1.6 ± 0.6	1.7 ± 0.6	n.s.
3	1.4 ± 0.7	1.7 ± 0.6	n.s.
4	1.4 ± 0.8	1.6 ± 0.7	n.s.
5	1.7 ± 0.9	1.8 ± 0.6	n.s.
6	1.6 ± 0.7	1.6 ± 0.7	n.s.
**7**	**0.8** ± **0.9**	**1.2** ± **0.8**	**<0.01** *
8	1.1 ± 0.9	1.3 ± 0.9	n.s.
9	0.8 ± 0.7	0.9 ± 0.7	n.s.

	Medial tibial plateau		
Area	Primary	Second look	*p* Value
1	1.7 ± 0.6	1.8 ± 0.5	n.s.
2	1.9 ± 0.3	2.0 ± 0.5	n.s.
3	2.0 ± 0.2	2.1 ± 0.2	n.s.
4	1.8 ± 0.5	1.9 ± 0.4	n.s.
5	1.9 ± 0.4	1.9 ± 0.3	n.s.

*Note*: Data are displayed as a mean standard deviation. The significance was determined with the use of the Wilcoxon signed‐rank test.

Abbreviations: ICRS, International Cartilage Research Society; n.s., not significant.

* Values in bold indicate statistical significance (*p* < 0.05).

ASI of the IPFP was not detected on preoperative MRI; however, it was observed postoperatively in 15 (40%) patients. When comparing arthroscopy and MRI changes in patients with and without ASI of the IPFP, the rate of ICRS grade worsening (≥2 grades) in the PF joint was significantly higher in patients with ASI of the IPFP than in those without (53% vs. 20%, *p* = 0.04) (Table 4). In patients with ASI of the IPFP, the ΔPAL in 90° knee flexion on MRI was significantly decreased postoperatively compared with that observed in patients without ASI of the IPFP (−1.5 mm vs. −0.2 mm; *p* < 0.01) (Table 4). The ICCs for intraobserver and interobserver correlations for PAL were 0.91 and 0.85, respectively.

**Table 4:** Comparison of the arthroscopic and radiographic findings between groups classified according to ASI of IPFP.

	ASI of IPFP (+) (*n* = 15)	ASI of IPFP (−) (*n* = 25)	*p* Value
**Arthroscopic findings**			
**ICRS worsening (≥2 grades) in PF (P/N)**	**8/7 (53%)**	**5/20 (20%)**	**0.04** *
ICRS worsening (≥2 grades) in MFC/MTP (P/N)	4/11 (26%)	9/16 (36%)	n.s.
Meniscal healing score, 0/1/2/3/4/5/6/7/8/9/10	0/0/0/0/1/1/4/2/5/1/1	0/0/0/0/0/1/2/9/7/4/1	n.s.
**MRI findings**			
**10° knee‐flexion MRI**			
**ASI of suprapatellar pouch (%)**	**13/2 (87%)**	**8/17 (32%)**	**<0.01** *
Preoperative patellar tendon length, mm	40.1 ± 4.9	41.8 ± 4.5	n.s.
1Y postoperative patellar tendon length, mm	40.4 ± 5.7	42.2 ± 4.5	n.s.
Δ patellar tendon length, mm	0.2 ± 2.3	0.4 ± 1.5	n.s.
Preoperative Insall Salvati, %	1.01 ± 0.1	1.04 ± 0.1	n.s.
1Y postoperative Insall Salvati, %	1.03 ± 0.1	1.04 ± 0.1	n.s.
Δ Insall Salvati, %	0.02 ± 0.1	0.01 ± 0.1	n.s.
Preoperative patellar tilt, deg	6.3 ± 2.5	5.0 ± 4.4	n.s.
1Y postoperative patellar tilt, deg	7.3 ± 3.1	4.8 ± 4.4	n.s.
Δ patellar tilt, deg	1.1 ± 1.8	−0.3 ± 2.5	n.s.
**90° knee‐flexion MRI**			
Preoperative PAL, mm	20.3 ± 4.6	21.2 ± 3.7	n.s.
1Y postoperative PAL, mm	18.7 ± 4.9	21.0 ± 3.6	n.s.
**Δ PAL, mm**	**−1.5** ± **0.7**	**−0.2** ± **0.3**	**<0.01** *

*Note*: Values are presented as mean ± standard deviation or number.

Abbreviations: 1Y, 1‐year; ASI, abnormal signal intensity; IPFP, infrapatellar fat pad; MFC, medial femoral condyle; MRI, magnetic resonance imaging; MTP, medial tibial plateau; n.s., not significant; PAL, patellar‐anterior cruciate ligament insertion length; PF, patellofemoral joint; P/N, positive/negative.

* Values in bold indicate statistical significance (*p* < 0.05).

All clinical scores, including the KOOS and VAS pain score, improved postoperatively in both groups. However, the postoperative VAS pain score was significantly higher in patients with ASI of the IPFP than in those without (Table 5).

**Table 5:** Comparison of preoperative and postoperative clinical scores between groups classified according to ASI of IPFP.

	ASI of IPFP (+) (*n* = 15)	ASI of IPFP (−) (*n* = 25)	*p* Value
KOOS‐pain			
Preoperative	63.0 ± 16.8	61.6 ± 15.8	n.s.
Postoperative	85.2 ± 12.6	87.8 ± 13.6	n.s.
*p* Value	<**0.01** *	<**0.01** *	
KOOS‐symptoms			
Preoperative	65.4 ± 18.7	67.3 ± 15.0	n.s.
Postoperative	79.8 ± 14.6	82.4 ± 13.9	n.s.
*p* Value	<**0.01** *	<**0.01** *	
KOOS‐ADL			
Preoperative	67.7 ± 15.0	68.5 ± 17.1	n.s.
Postoperative	86.9 ± 12.8	86.6 ± 14.9	n.s.
*p* Value	<**0.01** *	<**0.01** *	
KOOS‐Sport/Rec			
Preoperative	20.0 ± 19.4	27.6 ± 28.3	n.s.
Postoperative	45.7 ± 25.9	52.2 ± 34.5	n.s.
*p* Value	<**0.01** *	<**0.01** *	
KOOS‐QOL			
Preoperative	27.3 ± 19.9	36.0 ± 22.6	n.s.
Postoperative	61.8 ± 23.7	65.4 ± 25.8	n.s.
*p* Value	<**0.01** *	<**0.01** *	
**Pain score (VAS, 0–100)**			
Preoperative	31.1 ± 23.7	36.6 ± 25.5	n.s.
**Postoperative**	**13.4** ± **14.7**	**7.3** ± **13.6**	**0.04** *
*p* Value	<**0.01** *	<**0.01** *	

*Note*: Values are presented as mean ± standard deviation or number and first‐third quartiles.

Abbreviations: ADL, activities of daily living; ASI, abnormal signal intensity; IPFP, infrapatellar fat pad; KOOS, knee injury and osteoarthritis outcome score; n.s., not significant; QOL, quality of life; Sport/Rec, sport and recreation; VAS, Visual analogue scale.

* Values in bold indicate statistical significance (*p* < 0.05).

Among risk factors for ASI, patients with ASI of the IPFP had a significantly higher rate of 2‐week postoperative immobilization than those without (67% vs. 20%, *p* < 0.01) (Table 6). Logistic regression analysis revealed that postoperative rehabilitation was a significant risk factor for ASI of the IPFP (Table 7).

**Table 6:** Comparison of patient demographics between groups classified according to ASI of IPFP.

	ASI of IPFP (+) (*n* = 15)	ASI of IPFP (−) (*n* = 25)	*p* Value
Sex (male/female)	4/11	6/19	n.s.
Age (years)	65.2 ± 7.9	63.3 ± 9.2	n.s.
Body mass index (kg/m^2)^	24.3 ± 2.4	25.8 ± 2.4	n.s.
Femorotibial angle (deg)	177.7 ± 2.4	177.3 ± 2.2	
Preoperative Kellgren–Lawrence grade (0:1:2)	0:7:8	0:14:11	n.s.
**Postoperative immobilization (2 weeks/1 week)**	**10/5 (67%)**	**5/20 (20%)**	**<0.01** *
Surgical technique (TSS/TSS + PM/TCS/TCS + PA)	5/4/2/4	5/5/7/8	n.s.
Diabetes (P/N)	1/14	3/22	n.s.
Thyroid dysfunction (P/N)	1/14	1/24	n.s.

*Note*: Values are presented as mean ± standard deviation or number.

Abbreviations: ASI, abnormal signal intensity; IPFP, infrapatellar fat pad; n.s., not significant; PA, posterior anchoring; PF, patellofemoral joint; PM, posteromedial pullout; P/N, positive/negative; TCS, two cinch stitches; TSS, two simple stitches.

* Values in bold indicate statistical significance (*p* < 0.05).

**Table 7:** Logistic regression analysis of the factors related to ASI of IPFP.

			95% CI	
Variables	*p* Value	Odds ratio	Lower	Upper
Age	0.76	1.02	0.91	1.13
Body mass index	0.13	0.72	0.47	1.10
Sex (male, female)	0.64	0.65	0.10	4.04
**Postoperative immobilization (2 weeks, 1 week)**	**0.01** *	**0.12**	**0.02**	**0.62**
Preoperative Insall Salvati	0.49	0.08	0.00	114.0

Abbreviations: ASI, abnormal signal intensity; CI, confidence interval; IPFP, infrapatellar fat pad.

* Values in bold indicate statistical significance (*p* < 0.05).

## DISCUSSION

The most important finding of this study cartilage deterioration not only in the femorotibial joint but also in the PF joint after MMPR repair. Moreover, ICRS grade worsening in the PF joint was observed more frequently in patients with ASI of the IPFP, and the PAL in knee flexion shortened postoperatively in those with ASI of the IPFP.

IPFP fibrosis could be a cause of knee stiffness, anterior knee pain and progression of PF degeneration after knee surgery, such as ACL reconstruction (ACLR) [25, 29]. Hoon et al. reported the following three types of IPFP fibrosis: focal fibrosis (64%), complete fibrosis (28%) and diffuse and infiltrated fibrosis (6.5%); Nakagawa et al. recently reported severe fibrosis with infiltration in the IPFP in seven of 36 patients (19%) after ACLR, observing that severe fibrosis could decrease postoperative clinical scores [25]. In the present study, infiltrated severe fibrosis was not found after MMPR repair, possibly owing to less inflammation in the knee joint caused by shaving of the anterior interval or small bone tunnel aperture compared with ACLR. However, the rate of ASI of the IPFP (38%, 15/40 patients) was higher than that reported in previous ACLR studies [25, 29], indicating that PF cartilage degeneration could progress postoperatively in middle‐aged and older patients after MMPR repair. However, these studies did not assess lesions of the chondral damage, while in the current study, PF degeneration mostly occurred in the superior and medial aspect of the patella and the central trochlea.

**Figure 4:**
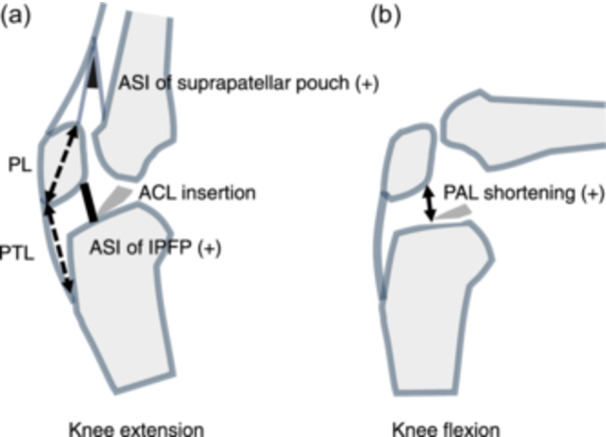
Illustrations of abnormal signal intensity (ASI) of the infrapatellar fat pad (IPFP). (a) Continuous ASI of IPFP in knee extension. Concomitant ASI of the suprapatellar pouch is frequently confirmed. The Insall‐Salvati (PTL/PL) ratio did not change postoperatively. (b) PAL shortened postoperatively in knee flexion with ASI of the IPFP. ACL, anterior cruciate ligament; PAL, patellar‐anterior cruciate ligament insertion length; PL, patellar length; PTL, patellar tendon length.

The Insall‐Salvati and patellar tendon lengths remained unchanged postoperatively; PAL shortening in knee flexion was found in patients with the ASI of the IPFP (Figure 4). Furthermore, ASI of the suprapatellar pouch frequently coexisted with ASI of the IPFP. These findings suggest fibrosis of the IPFP and that the suprapatellar pouch could restrict normal knee kinematics. Previous biomechanical studies have revealed that fibrosis in the anterior interval and suprapatellar pouch increases PF contact force during knee flexion [1, 20]. Another study on knees with arthrofibrosis after ACLR revealed abnormal patellar movement (medial tilt, flexion and inferior shift) during knee flexion compared with that in the contralateral intact knee [31]. Our findings of delta PAL shortening and the area of PF worsening are consistent with those of these previous reports.

Although differences between the two types of rehabilitation protocol are small, 2 weeks of immobilization may be a risk factor for IPFP fibrosis. Postoperative rehabilitation should balance the risk of meniscus‐healing failure due to early mobilization with the risk of complications, such as adhesions and muscle atrophy, resulting from prolonged immobilization. Meniscus‐healing status was similar between the groups with or without ASI of the IPFP; however, the pain score was greater in patients with ASI of the IPFP than in those without. Previously, prolonged postoperative restriction of partial or full weight‐bearing was common practice; partial weight‐bearing typically started at 2–6 weeks and full weight‐bearing at 6–10 weeks postoperatively [4, 8, 15, 18, 23]. However, overprotected weight‐bearing and ROM can exacerbate postoperative muscle weakness, which may require 6–12 months of intense rehabilitation for normalization [30]. A recent retrospective study revealed that postoperative quadriceps muscle strength is associated with clinical scores and postoperative progression of medial meniscal extrusion after MMPR repair [14]. Therefore, minimizing quadriceps‐strength loss in the early postoperative period is important. The strength of other muscles, such as the hip abductor and hip rotator, along with muscle tightness of the iliotibial band and hamstrings, is important in preventing the progression of PF degeneration [21]. The effectiveness of extra‐articular rehabilitation should be investigated in future studies.

This study has some limitations. First, the retrospective nature of this study may have led to a selection bias. Second, follow‐up was limited to only 1 year and a longer follow‐up could reveal whether the observed PF degeneration progresses or stabilizes, which is critical for understanding the full impact of MMPR repair on long‐term knee health. A recent systematic review showed that femorotibial cartilage degeneration progresses by at least 1 grade on MRI scans in 23% of patients at a mean follow‐up of 31.6 months even after MMPR repair [3]. Further research is required to elucidate the association between PF degeneration and medial femorotibial degeneration. Third, PF‐specific scores such as the Kujala or HSS Patella scores were not assessed in this study. This may lead to an underestimation of the pathology of the PF joint, although KOOS is frequently used after meniscus repair [2]. Fourth, different types of suture configurations were utilized in this study. Suture configuration was changed aiming to enhance initial meniscus stability according to the timing of the surgery in this study; however, biomechanically, no suture configuration could achieve the strength of a native meniscus root (359–678 N) [7, 13, 22] and the distribution of the four suturing techniques and intraoperative meniscus healing status at 1‐year second‐look were similar between the groups with or without ASI of IPFP (Table 6). Therefore, we assume that the difference in suture configuration had little effect on PF degeneration. Fifth, the impact of the two different rehabilitation protocols on factors such as quadriceps muscle strength was not investigated. Sixth, the extent of arthroscopic debridement of the IPFP during primary surgery may have affected the results; however, the same orthopaedic surgeon performed all surgical procedures in this study; therefore, this limitation is unlikely to have had an impact on the study findings.

## CONCLUSIONS

PF chondral degeneration progressed after MMPR repair with ASI of the IPFP. Therefore, reducing the ASI of the IPFP is important to prevent the progression of femorotibial and PF cartilage degeneration. Unnecessary immobilization may increase the risk of fibrosis of the IPFP; however, large‐scale prospective studies are required to determine the optimal rehabilitation protocol. The presence of ASI in the IPFP may negatively affect the postoperative VAS pain score. Longer postoperative immobilization may be a risk factor for ASI of the IPFP.

## AUTHOR CONTRIBUTIONS

Masanori Tamura and Takayuki Furumatsu designed the study. Masanori Tamura and Takayuki Furumatsu contributed to the analysis and interpretation of data. All authors contributed to data collection and interpretation and critically reviewed the manuscript.

## CONFLICT OF INTEREST STATEMENT

The authors declare no conflicts of interest.

## ETHICS STATEMENT

This study was approved by the Institutional Review Board of Okayama University (ethical approval number: N1857). Written informed consent was obtained from all study participants.

## Supporting information

Supporting information.

## Data Availability

The data sets generated and analyzed during the current study are available from the corresponding author upon reasonable request.

## References

[1] Ahmad, C.S. , Kwak, S.D. , Ateshian, G.A. , Warden, W.H. , Steadman, J.R. & Mow, V.C. (1998) Effects of patellar tendon adhesion to the anterior tibia on knee mechanics. The American Journal of Sports Medicine, 26, 715–724. Available from: 10.1177/03635465980260051901 9784821

[2] Bourlez, J. , Canovas, F. , Duflos, C. & Dagneaux, L. (2019) Are modern knee outcomes scores appropriate for evaluating patellofemoral degeneration in osteoarthritis? Evaluation of the ceiling and floor effects in knee outcomes scores. Orthopaedics & Traumatology: Surgery & Research, 105, 599–603. Available from: 10.1016/j.otsr.2019.01.018 30935814

[3] Chang, P.S. , Radtke, L. , Ward, P. & Brophy, R.H. (2022) Midterm outcomes of posterior medial meniscus root tear repair: a systematic review. The American Journal of Sports Medicine, 50, 545–553. Available from: 10.1177/0363546521998297 33780278

[4] Cho, J.H. & Song, J.G. (2014) Second‐look arthroscopic assessment and clinical results of modified pull‐out suture for posterior root tear of the medial meniscus. Knee Surgery & Related Research, 26, 106–113. Available from: 10.5792/ksrr.2014.26.2.106 24944976 PMC4061404

[5] Dwyer, T. , Martin, C.R. , Kendra, R. , Sermer, C. , Chahal, J. , Ogilvie‐Harris, D. et al. (2017) Reliability and validity of the arthroscopic international cartilage repair society classification system: correlation with histological assessment of depth. Arthroscopy: The Journal of Arthroscopic & Related Surgery, 33, 1219–1224. Available from: 10.1016/j.arthro.2016.12.012 28162918

[6] Dzidzishvili, L. , Calvo, E. , & López‐Torres II, I.I. (2023) Medial meniscus posterior root repair reduces but does not avoid histologic progression of osteoarthritis: randomized in vivo experimental study in a rabbit model. The American Journal of Sports Medicine, 51, 2964–2974. Available from: 10.1177/03635465231188527 37589243

[7] Ellman, M.B. , LaPrade, C.M. , Smith, S.D. , Rasmussen, M.T. , Engebretsen, L. , Wijdicks, C.A. et al. (2014) Structural properties of the meniscal roots. The American Journal of Sports Medicine, 42, 1881–1887. Available from: 10.1177/0363546514531730 24799425

[8] Furumatsu, T. , Hiranaka, T. , Okazaki, Y. , Kintaka, K. , Kodama, Y. , Kamatsuki, Y. et al. (2022) Medial meniscus posterior root repairs: a comparison among three surgical techniques in short‐term clinical outcomes and arthroscopic meniscal healing scores. Journal of Orthopaedic Science, 27, 181–189. Available from: 10.1016/j.jos.2020.11.013 33581924

[9] Furumatsu, T. , Miyazawa, S. , Fujii, M. , Tanaka, T. , Kodama, Y. & Ozaki, T. (2019) Arthroscopic scoring system of meniscal healing following medial meniscus posterior root repair. International Orthopaedics, 43, 1239–1245. Available from: 10.1007/s00264-018-4071-z 30069591

[10] Guilbert, S. , Chassaing, V. , Radier, C. , Hulet, C. , Rémy, F. , Chouteau, J. et al. (2013) Axial MRI index of patellar engagement: a new method to assess patellar instability. Orthopaedics & Traumatology: Surgery & Research, 99, S399–S405. Available from: 10.1016/j.otsr.2013.10.006 24268843

[11] Hantouly, A.T. , Aminake, G. , Khan, A.S. , Ayyan, M. , Olory, B. , Zikria, B. et al. (2024) Meniscus root tears: state of the art. International Orthopaedics, 48, 955–964. Available from: 10.1007/s00264-024-06092-w 38261073 PMC10933189

[12] Jackson, G.R. , Warrier, A.A. , Wessels, M. , Khan, Z.A. , Obioha, O. , McCormick, J.R. et al. (2024) A systematic review of adverse events and complications after isolated posterior medial meniscus root repairs. The American Journal of Sports Medicine, 52, 1109–1115. Available from: 10.1177/03635465231157758 37129097

[13] Jiang, E.X. , Everhart, J.S. , Abouljoud, M. , Kirven, J.C. , Magnussen, R.A. & Kaeding, C.C. et al. (2019) Biomechanical properties of posterior meniscal root repairs: a systematic review. Arthroscopy: The Journal of Arthroscopic & Related Surgery, 35, 2189–2206.e2. Available from: 10.1016/j.arthro.2019.01.018 30979628

[14] Kawada, K. , Furumatsu, T. , Fukuba, M. , Tamura, M. , Higashihara, N. , Okazaki, Y. et al. (2023) Increased quadriceps muscle strength after medial meniscus posterior root repair is associated with decreased medial meniscus extrusion progression. BMC Musculoskeletal Disorders, 24, 727. Available from: 10.1186/s12891-023-06858-0 37700279 PMC10496236

[15] Kim, C.W. , Lee, C.R. , Gwak, H.C. , Kim, J.H. , Park, D.H. , Kwon, Y.U. et al. (2019) Clinical and radiologic outcomes of patients with lax healing after medial meniscal root repair: comparison with subtotal meniscectomy. Arthroscopy: The Journal of Arthroscopic & Related Surgery, 35, 3079–3086. Available from: 10.1016/j.arthro.2019.05.051 31629584

[16] Kim, J.Y. , Bin, S.I. , Kim, J.M. , Lee, B.S. , Oh, S.M. & Cho, W.J. (2019) A novel arthroscopic classification of degenerative medial meniscus posterior root tears based on the tear gap. Orthopaedic Journal of Sports Medicine, 7, 2325967119827945. Available from: 10.1177/2325967119827945 30911565 PMC6423685

[17] Krych, A.J. , Johnson, N.R. , Mohan, R. , Dahm, D.L. , Levy, B.A. & Stuart, M.J. (2018) Partial meniscectomy provides no benefit for symptomatic degenerative medial meniscus posterior root tears. Knee Surgery, Sports Traumatology, Arthroscopy: Official Journal of the ESSKA, 26, 1117–1122. Available from: 10.1007/s00167-017-4454-5 28184957

[18] Lee, S.S. , Ahn, J.H. , Kim, J.H. , Kyung, B.S. & Wang, J.H. (2018) Evaluation of healing after medial meniscal root repair using second‐look arthroscopy, clinical, and radiological criteria. The American Journal of Sports Medicine, 46, 2661–2668. Available from: 10.1177/0363546518788064 30118319

[19] Loyst, R.A. , Palhares, G. , Hinkley, P. , Rizy, M. , Burge, A.J. , Gomoll, A.H. et al. (2023) Predilection of patellofemoral cartilage lesions in patients with posterior medial meniscal root lesions. Cartilage, 14, 407–412. Available from: 10.1177/19476035231184618 37496261 PMC10807735

[20] Mikula, J.D. , Slette, E.L. , Dahl, K.D. , Montgomery, S.R. , Dornan, G.J. , O'Brien, L. et al. (2017) Intraarticular arthrofibrosis of the knee alters patellofemoral contact biomechanics. Journal of Experimental Orthopaedics, 4, 40. Available from: 10.1186/s40634-017-0110-8 29260429 PMC5736518

[21] Mills, K. & Hunter, D.J. (2014) Patellofemoral joint osteoarthritis: an individualised pathomechanical approach to management. Best Practice & Research Clinical Rheumatology, 28, 73–91. Available from: 10.1016/j.berh.2014.01.006 24792946

[22] Mitchell, R. , Pitts, R. , Kim, Y.M. & Matava, M.J. (2016) Medial meniscal root avulsion: a biomechanical comparison of 4 different repair constructs. Arthroscopy: The Journal of Arthroscopic & Related Surgery, 32, 111–119. Available from: 10.1016/j.arthro.2015.07.013 26422709

[23] Moon, H.S. , Choi, C.H. , Jung, M. , Lee, D.Y. , Hong, S.P. & Kim, S.H. (2020) Early surgical repair of medial meniscus posterior root tear minimizes the progression of meniscal extrusion: 2‐year follow‐up of clinical and radiographic parameters after arthroscopic transtibial pull‐out repair. The American Journal of Sports Medicine, 48, 2692–2702. Available from: 10.1177/0363546520940715 32730732

[24] Murphy, S.N. , Brinkman, J.C. , Tummala, S.V. , Renfree, S.P. , Kemper, K.J. & Economopoulos, K.J. (2023) Outcomes after meniscal root repair in patients with and without advanced patellofemoral chondromalacia: comparison at 2‐year follow‐up. Orthopaedic Journal of Sports Medicine, 11, 23259671231193986. Available from: 10.1177/23259671231193986 37711507 PMC10498705

[25] Nakagawa, Y. , Tsuji, K. , Nakamura, T. , Katagiri, H. , Ozeki, N. , Shioda, M. et al. (2023) Association of infrapatellar fat pad fibrosis at 3 months after ACL reconstruction with short‐term clinical outcomes and inflammatory cytokine levels in the synovial fluid. Orthopaedic Journal of Sports Medicine, 11, 23259671231164122. Available from: 10.1177/23259671231164122 37123994 PMC10134128

[26] Ozeki, N. , Koga, H. & Sekiya, I. (2022) Degenerative meniscus in knee osteoarthritis: from pathology to treatment. Life, 12, 603. Available from: 10.3390/life12040603 35455094 PMC9032096

[27] Shabshin, N. , Schweitzer, M. , Morrison, W. & Parker, L. (2004) MRI criteria for patella alta and baja. Skeletal Radiology, 33, 445–450. Available from: 10.1007/s00256-004-0794-6 15221214

[28] Tamura, M. , Furumatsu, T. , Yokoyama, Y. , Kintaka, K. , Higashihara, N. , Kawada, K. et al. (2024) Assessing the frequency and effectiveness of various arthroscopic treatments in the management of symptomatic isolated medial meniscus injuries including medial meniscus posterior root tear: a retrospective observational cohort study. Acta Medica Okayama, 78, 21–27. Available from: 10.18926/AMO/66667 38419311

[29] Ueda, Y. , Matsushita, T. , Araki, D. , Kida, A. , Takiguchi, K. , Shibata, Y. et al. (2017) Factors affecting quadriceps strength recovery after anterior cruciate ligament reconstruction with hamstring autografts in athletes. Knee Surgery, Sports Traumatology, Arthroscopy, 25, 3213–3219. Available from: 10.1007/s00167-016-4296-6 27553297

[30] Venkatachalam, S. , Godsiff, S.P. & Harding, M.L. (2001) Review of the clinical results of arthroscopic meniscal repair. The Knee, 8, 129–133. Available from: 10.1016/S0968-0160(01)00061-8 11337239

[31] Zhang, L. , Wang, S. , Fan, S. , Ye, J. & Cai, B. (2021) Knee extensor mechanism strength and its relationship to patellofemoral kinematics in individuals with arthrofibrosis within 6 months after anterior cruciate ligament reconstruction. Journal of Sport Rehabilitation, 30, 1138–1143. Available from: 10.1123/jsr.2020-0468 34111841

[32] Zhang, X. , Furumatsu, T. , Hiranaka, T. , Okazaki, Y. , Xue, H. , Kintaka, K. et al. (2023) The stability of repaired meniscal root can affect postoperative cartilage status following medial meniscus posterior root repair. Journal of Orthopaedic Science, 28, 1060–1067. Available from: 10.1016/j.jos.2022.08.005 36089432

