# Supplementary material for: Progression of patellofemoral joint cartilage degeneration within 1 year after medial meniscus posterior root repair: A retrospective study
Source: J Exp Orthop. 2025 Apr 1;12(2):e70139. doi: 10.1002/jeo2.70139 (PMC11959506; doi:10.1002/jeo2.70139)
Supplement: Supplementary file 1 — Supporting information. [file JEO2-12-e70139-s001.pptx]

## Slide 1
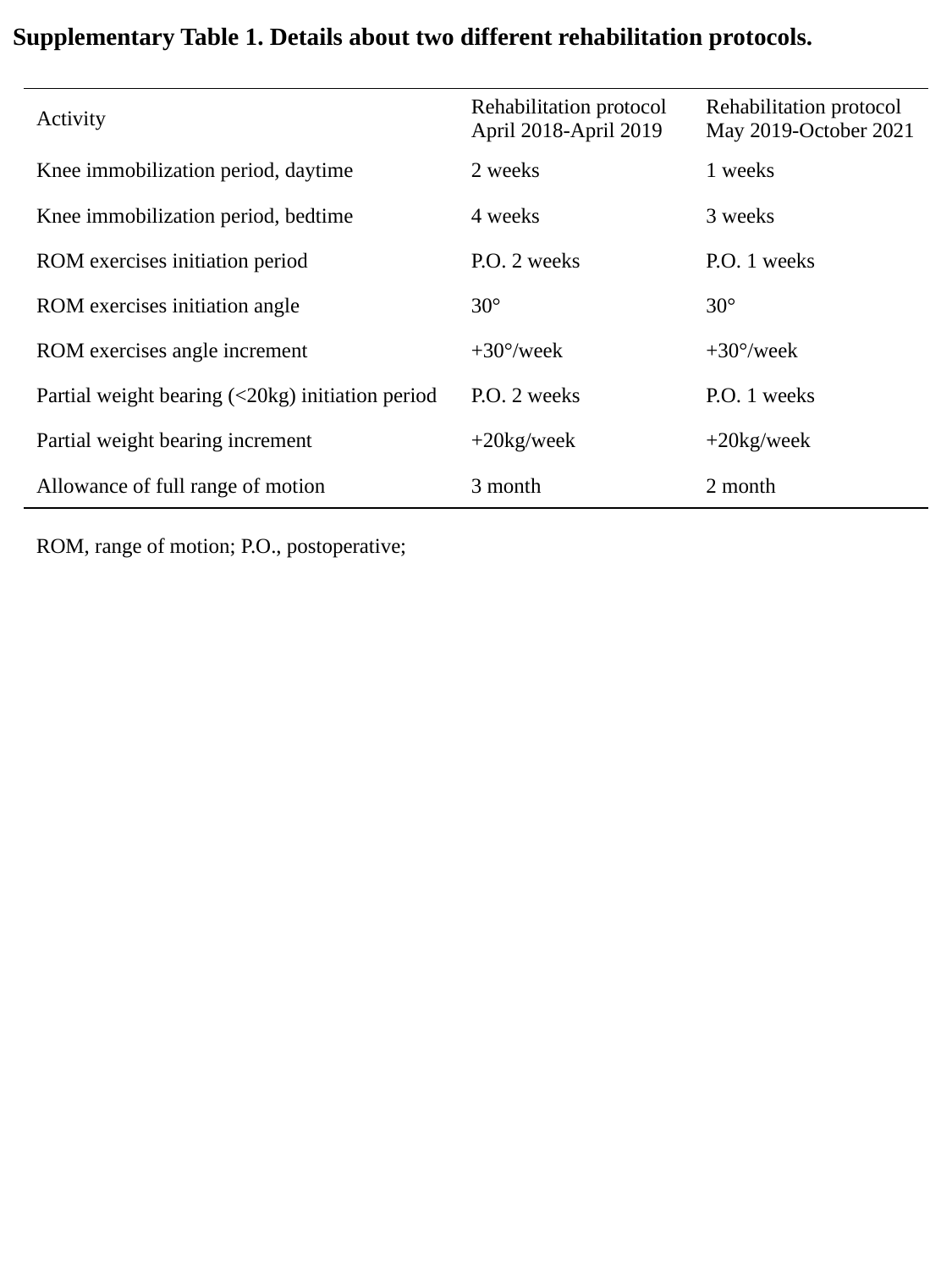

Supplementary Table 1. Details about two different rehabilitation protocols.
| Activity | Rehabilitation protocol April 2018-April 2019 | Rehabilitation protocol May 2019-October 2021 |
| --- | --- | --- |
| Knee immobilization period, daytime | 2 weeks | 1 weeks |
| Knee immobilization period, bedtime | 4 weeks | 3 weeks |
| ROM exercises initiation period | P.O. 2 weeks | P.O. 1 weeks |
| ROM exercises initiation angle | 30° | 30° |
| ROM exercises angle increment | +30°/week | +30°/week |
| Partial weight bearing (<20kg) initiation period | P.O. 2 weeks | P.O. 1 weeks |
| Partial weight bearing increment | +20kg/week | +20kg/week |
| Allowance of full range of motion | 3 month | 2 month |
ROM, range of motion; P.O., postoperative;
